# Reflecting on the utility of standardized uptake values on ^18^F-FDG PET in nasopharyngeal carcinoma

**DOI:** 10.1186/s12885-022-09626-w

**Published:** 2022-05-05

**Authors:** Xiufang Qiu, Haixia Wu, Ting Xu, Shihan Xie, Ziqing You, Yixin Hu, Yinghong Zheng, Zewei Liang, Chaoxiong Huang, Li Yi, Li Li, Jing Liu, Zhaodong Fei, Chuanben Chen

**Affiliations:** 1 College of Clinical Medicine for Oncology, Fujian Medical University, Fujian Cancer Hospital, Fujian, People’s Republic of China; 2 Fujian Medical University, Fujian, People’s Republic of China; 3 Department of Radiation Oncology, Fujian Medical University Cancer Hospital, Fujian Cancer Hospital, Fuzhou, 350014 Fujian People’s Republic of China

**Keywords:** Nasopharyngeal carcinoma, ^18^F-fluorodeoxyglucose positron-emission tomography, Standardized uptake values

## Abstract

**Background:**

To rethink the clinical significance of standardized uptake values (SUVs) of nasopharyngeal carcinoma (NPC) on ^18^F-fluorodeoxyglucose (^18^F-FDG) positron-emission tomography (PET).

**Methods:**

We retrospectively reviewed 369 NPC patients who underwent pretreatment ^18^F-FDG PET. The predictive value of the SUVmax of the primary tumor (SUVmax-t) and regional lymph nodes (SUVmax-n) was evaluated using probability density functions. Receiver operating characteristic curves were used to determine optimal cutoffs for the SUVmax-n/SUVmax-t ratio (NTR). Kaplan–Meier and Cox regression analyses were used to assess survival.

**Results:**

The optimal SUVmax-t and SUVmax-n cutoffs were 7.5 and 6.9, respectively. High SUVmax-t and SUVmax-n were related to local and regional recurrence, respectively. Patients with low SUVmax had better 3-year overall survival (OS). To avoid cross-sensitization of cutoff points, we stratified patients with high SUVmax into the low and high NTR groups. The 3-year distant metastasis-free survival (DMFS; 92.3 vs. 80.6%, *P* = 0.009), progression-free survival (PFS; 84.0 vs. 67.7%, *P* = 0.011), and OS (95.9 vs. 89.2%, *P* = 0.002) significantly differed between the high vs. low NTR groups for patients with high SUVmax. Multivariable analysis showed that NTR was an independent prognostic factor for DMFS (hazard ratio [HR]: 2.037, 95% CI: 1.039–3.992, *P* = 0.038), PFS (HR: 1.636, 95% CI: 1.021–2.621, *P* = 0.041), and OS (HR: 2.543, 95% CI: 1.214–5.325, *P* = 0.013).

**Conclusion:**

High SUVmax was associated with NPC recurrence. NTR is a potential prognosticator for DMFS, suggesting that heterogeneity in the pretreatment ^18^F-FDG uptake between the primary tumor and lymph nodes is associated with high invasion and metastatic potential.

**Supplementary Information:**

The online version contains supplementary material available at 10.1186/s12885-022-09626-w.

## Introduction

Nasopharyngeal carcinoma (NPC) is an epithelial malignant tumor prevalent in East and Southeast Asia [1]. According to Global Cancer Statistics, an estimated 133,000 new cases of NPC were diagnosed in 2020 worldwide [2]. Radiotherapy or concurrent chemoradiotherapy is widely used as the standard treatment for NPC [3].

The American Joint Committee on Cancer (AJCC) TNM staging system is used globally to predict the prognosis and guide the treatment of NPC [4]. However, this staging system is largely based on anatomic imaging, which has limitations in terms of evaluating the aggressiveness of tumors. Owing to this, NPC patients with the same TNM stage can have substantial differences in prognosis yet receive similar treatments; hence, solely relying on the current anatomic imaging-based staging system is insufficient to accurately predict the prognosis of NPC patients [5]. Optimizing the conventional staging system and quantifying the recurrence risk are required to enable individualized therapy for NPC.

Some retrospective studies have indicated that the standardized uptake value (SUV) of ^18^F-fluorodeoxyglucose positron-emission tomography (^18^F-FDG PET) is useful for risk stratification and prognostication in NPC [6–11] Table 1. Although the optimal SUV cutoff points for NPC are still debated, their prognostic value cannot be denied. Hence, adopting the advantages of the previous research, the present study aimed to determine the association between SUVs on pretreatment ^18^F-FDG PET and prognosis in patients with NPC.

**Table 1:** Previous studies on SUVmax in nasopharyngeal carcinoma

	Risk factors	cut-off	Indicator	case
Chan SC [6]	SUVmax-t	12	5-year DRFS	65
Chan WK [7]	SUVmax-t SUVmax-n	7.5 6.5	2-year DFS	46
Cho H [8]	NLR-H	5.70	1-year DMFS	51
Hung TM [9]	NTR SUVmax-n	0.9181 7.4	5-year DMFS	437
Lee SJ [10]	SUVmax-n	13.4	3-year OS, DFS	53
Jeong Y [11]	SUVmax-t T–SUVpeak N(f)–SUVmax N(f)–SUVpeak	8.0 10.2 10.6 8.5	5-year DMFS	73

*Abbreviations*: *SUVmax-t* standardized uptake value of the primary tumor, *SUV max-n* the highest standardized uptake value of neck lymph nodes, *NLR-H* node-to-liver ratio with the highest up-take, *NTR* SUVmax-n/SUVmax-t ratio, *T-SUVpeak* peak standardized uptake value of the primary tumor, *N(f)–SUVmax* the SUVmax of the farthest lymph node station, *N(f)–SUVpeak* the SUVpeak of the farthest lymph node station

## Materials and methods

### Patients

In this retrospective study, we enrolled 369 NPC patients who had been newly diagnosed with NPC and underwent complete treatment in our cancer center between January 2012 and June 2017. Patients were consecutively recruited if they met the following inclusion criteria: (i) biopsy-proven primary NPC, (ii) pretreatment whole-body ^18^F-FDG PET/CT, (iii) radical treatment, (iv) age between 18 and 70 years, (v) complete medical history and clinical information, including physical examination, adequate clinical examination, and laboratory data, (vi) absence of distant metastasis before or during treatment, and (vii) no evidence of another primary carcinoma or other concomitant fatal disease. Patients who did not fulfill all the listed criteria were excluded from the study. All patients were restaged according to the 8th edition of the AJCC staging system. This study was approved by the ethics committee of Fujian Cancer Hospital (No. YKT2020-011-01).

### ^18^F-FDG PET/CT imaging

PET/CT scanning was performed using a Gemini TF 64 PET/CT scanner (Philips, The Netherlands) and the ^18^F-FDG was manufactured by HM-10 cyclotron with >95% radiochemical purity [12]. Before ^18^F-FDG PET/CT scanning, all patients fasted ≥6 hours to maintain serum blood glucose level of 3.9 ~ 6.5 mmol/L. Then ^18^F-FDG was intravenously administered at a dose of 148 to 296 MBq. Patients rested for 40 to 60 minutes in a dimly lit room before PET/CT scan. The CT scanning was from head to proximal thigh with the following acquisition parameters: 140 kV; 2.5 mA; matrix 512 ×512; and scan slice thickness 4 mm. The reconstructed PET images were obtained after applying the CT images for attenuation correction.

The ^18^F-FDG SUV was based on the region of interest (ROI) of tumor lesions. It was calculated as the decay-corrected tissue activity (nCi/mL) divided by the injected dose of FDG (nCi) and the patient’s body weight (g) [12]. SUVmax-t was defined as the maximum SUV of the primary tumor, and SUVmax-n was defined as the highest SUV of the regional lymph nodes. The lymph node-to-primary tumor SUV ratio (NTR), which was the ratio of SUVmax-n/SUVmax-t, was also assessed in this study.

### Chemotherapy

All chemotherapy regimens were administered according to a previously described protocol [13]. Patients with stage I disease received radiotherapy alone. Patients with stage II disease were administered radiotherapy along with 2–3 cycles of concurrent chemotherapy using a cisplatin-based regimen. Patients with stage III–IVA disease underwent 2–3 cycles of neoadjuvant chemotherapy prior to radiotherapy.

### Radiotherapy

All patients received intensity-modulated radiotherapy (IMRT), and the target volume and dose of radiotherapy were calculated using a previously described treatment protocol [13, 14]. In brief, the planning target volumes obtained for the primary gross tumor volume or gross tumor volume in the involved lymph nodes were exposed to a total dose of 70 Gy in 31–35 fractions. A total dose of 60 Gy was administered to the planning target volume for high-risk clinical target volumes. The corresponding dose to the planning target volume for potentially involved low-risk clinical target volumes and the clinical target volume of the neck nodal regions was 54 Gy, in total.

### Follow-up and clinical endpoints

The patients were examined every 3 months in the first 2 years, every 6 months in the following 3–5 years, and annually thereafter until death. The following endpoints were evaluated: local recurrence-free survival (LRFS, defined as time from diagnosis to local recurrence), regional recurrence-free survival (RRFS, time from diagnosis to regional recurrence), distant metastasis-free survival (DMFS, time from diagnosis to first distant metastasis), progression-free survival (PFS, time from diagnosis to disease progression or death from any cause), and overall survival (OS, time from diagnosis to death for any cause).

### Statistical analysis

All statistical analyses were performed using IBM SPSS statistical software, version 26.0 and R software, version 4.1.1. The best cutoff values were determined using receiver operating characteristic (ROC) curve analysis. Violin plots, Kaplan–Meier curves, and correlation plots were created using Hiplot (https://hiplot.com.cn). Multivariate analysis was carried out to identify the prognostic factors influencing PFS, OS, LRFS, RRFS, and DMFS. The Cox proportional hazards regression model was used for the multivariate analysis, and the results were presented as estimated hazard ratios (HRs) with 95% confidence intervals (CIs). Tests were two-sided, and *P* values < 0.05 were regarded as statistically significant.

## Results

### Patients’ characteristics and outcomes

The characteristics of the patients are summarized in Supplementary Table S1. This study included a total of 369 patients with a median age of 47 years (range, 19–70 years). The median follow-up time was 51 months (range, 4–105 months). At the end of follow-up, 33 patients (8.9%) had died, and 48 patients (13%) had experienced disease relapse in the form of local recurrence (34 patients, 9.2%), regional failure (20 patients, 5.4%), and distant metastasis (47 patients, 12.7%). The 3-year LRFS, RRFS, DMFS, PFS, and OS rates were 93.2%, 96.2%, 88.9%, 81.3%, and 94.6%, respectively.

### SUVmax and clinical stage

**Fig. 1:**
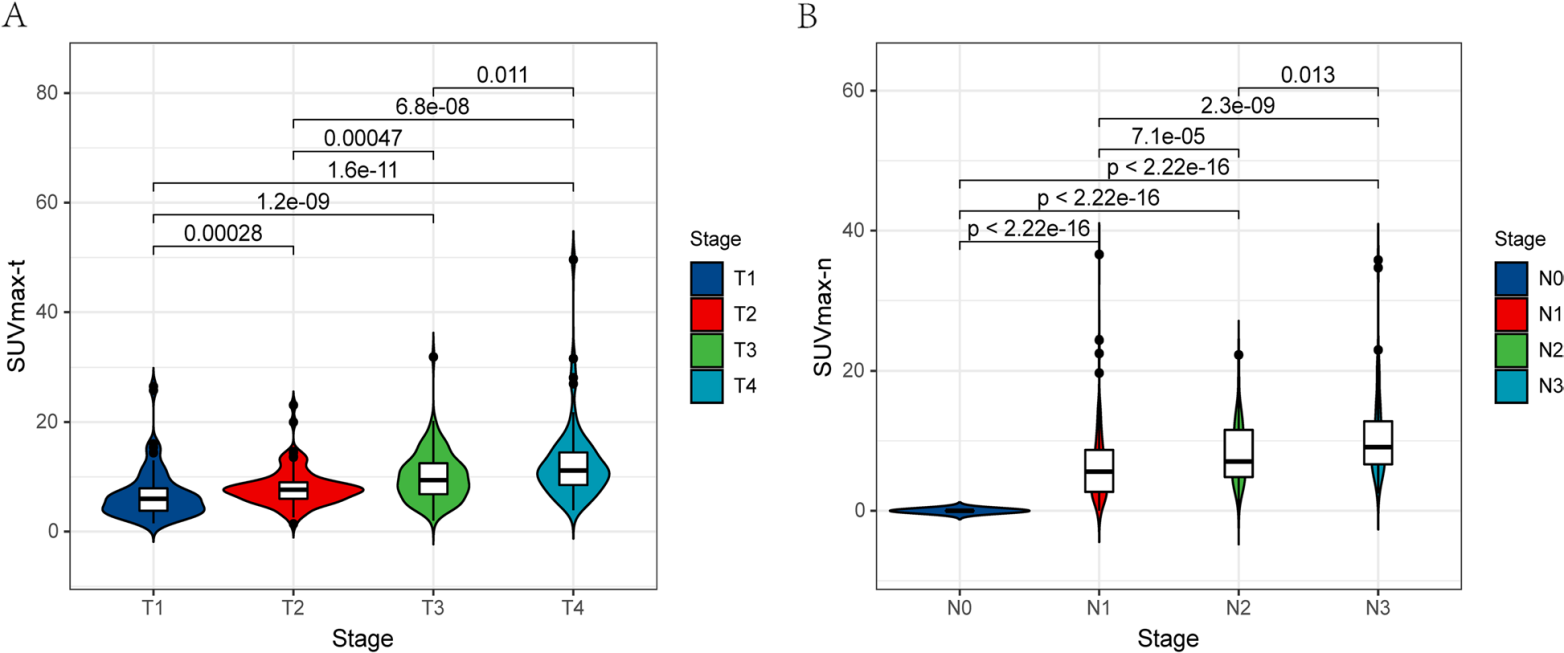
Violin plots. **A** Distribution of the SUVmax-t under T stage; **B** Distribution of the SUVmax-n under N stage

The SUVmax-t and SUVmax-n increased with the T and N stage, respectively (Fig. 1). When the values were distributed according to the disease stage, we found that the SUVmax-t data were more concentrated than the SUVmax-n data. The median SUVmax-t for the T1, T2, T3, and T4 stages were 6.2, 7.7, 9.4, and 11.2, respectively. The median SUVmax-n for the N0, N1, N2, and N3 stages were 1.6, 5.6, 7.1, and 9.1, respectively. The SUVmax-t and SUVmax-n significantly differed between different T stages and between different N stages, respectively (*P* < 0.001, Fig. 1). However, the SUVmax-t was not significantly correlated with the SUVmax-n (r = 0.13, *P* < 0.001, Supplementary Figure S1).

### Optimal cutoff points for SUVmax-t and SUVmax-n

**Fig. 2:**
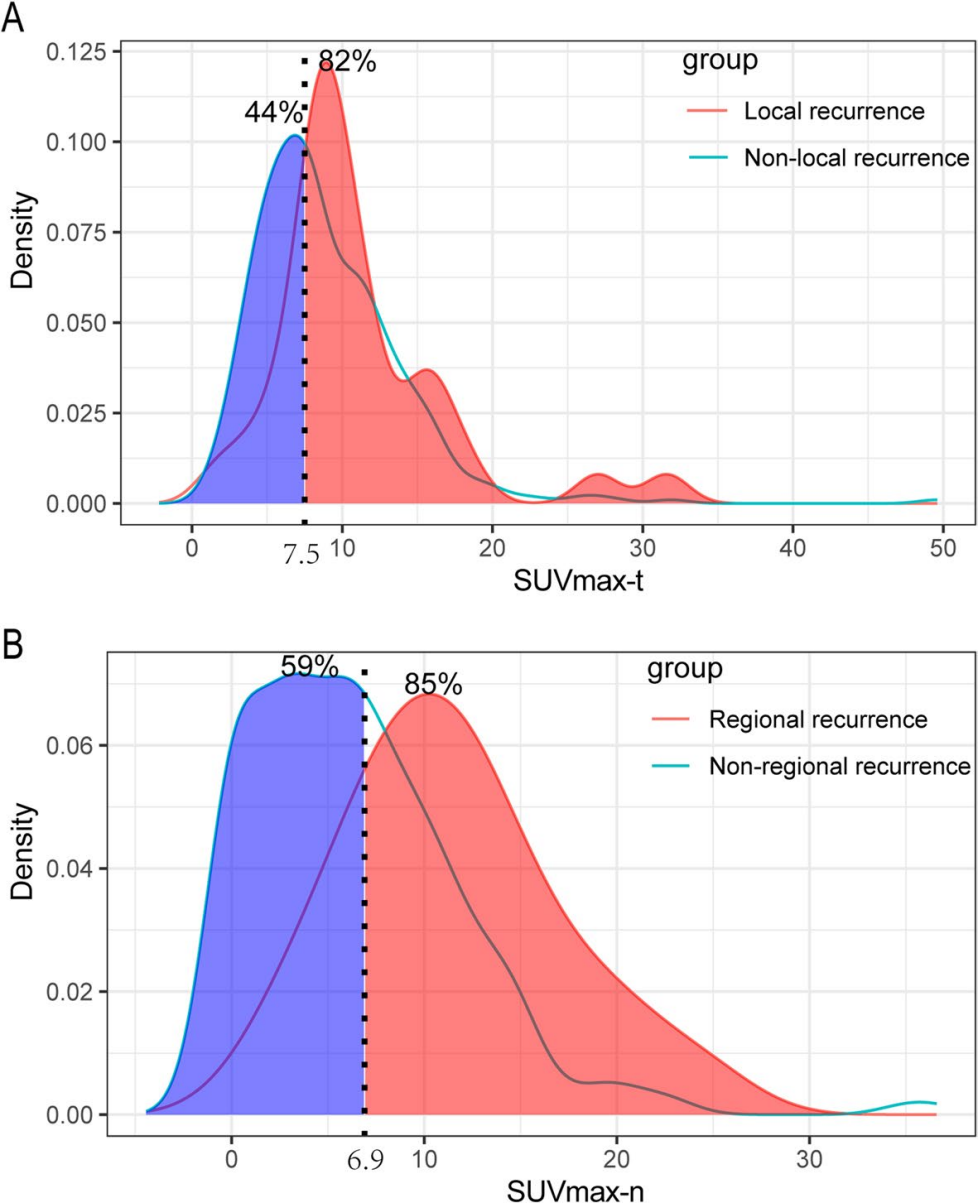
Probability density functions. **A** the SUVmax-t for predicting local recurrence; **B** the SUVmax-n for predicting regional recurrence

The mean SUVmax-t of the primary tumor and the mean SUVmax-n of cervical lymph node metastases were 9.2 ± 5.2 (range, 1.4–49.6) and 6.8 ± 5.8 (range, 0–36.6), respectively. The optimal cutoff SUVmax-t for predicting local recurrence was 7.5 (area under the curve [AUC] = 0.627, *P* = 0.015; Fig. 2). The optimal cutoff SUVmax-n for predicting regional recurrence was 6.9 (AUC = 0.757, *P* < 0.001). With an SUVmax-t threshold of 7.5, we could correctly identify approximately 82% of the patients with local recurrence. Moreover, at this cutoff, we could also identify approximately 44% of the patients with no risk of local recurrence (*P* < 0.001, Fig. 2A). With an SUVmax-n threshold of 6.9, we could correctly identify approximately 85% of the patients with regional recurrence and approximately 59% of the patients with no risk of regional recurrence (*P* < 0.001, Fig. 2B).

To control for potential confounders, we built a multivariable Cox proportional hazards model including all relevant variables. The results showed that SUVmax-t was an independent predictor of LRFS (HR = 3.741, 95% CI: 1.489–9.396, *P* = 0.005), while SUVmax-n was a risk factor for RRFS (HR = 3.238, 95% CI: 1.103–9.505, *P* = 0.033; Supplementary Table S2).

### SUVmax and prognosis

Considering the above results, we stratified patients into 4 groups: (a) LL group, low SUVmax-n (≤6.9) and low SUVmax-t (≤7.5), (b) LH group, low SUVmax-n (≤6.9) and high SUVmax-t (>7.5), (c) HL group, high SUVmax-n (>6.9) and low SUVmax-t (≤7.5), and (d) HH group, high SUVmax-n (>6.9) and high SUVmax-t (>7.5). To better explain the results, we demonstrated several representative ^18^F-FDG PET images for each subgroup (Supplementary Figure S2, S3, S4 and S5).

**Fig. 3:**
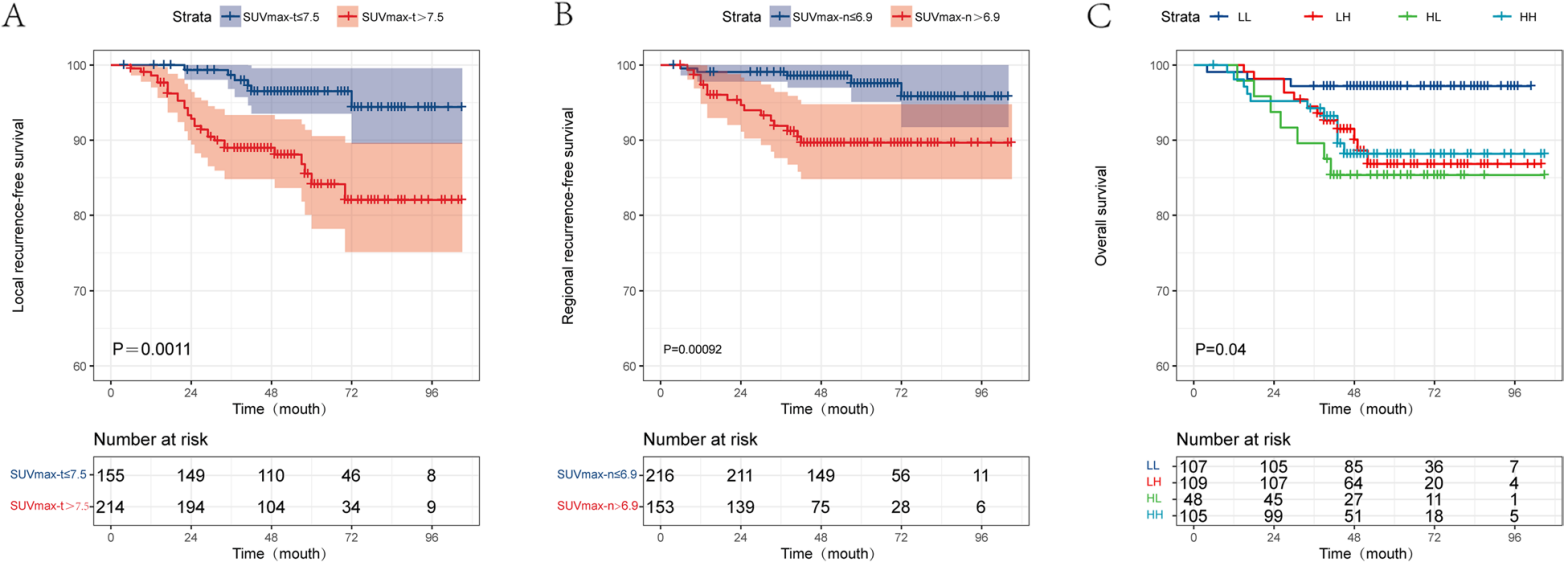
Kaplan-Meier curves by high and low SUVmax value. **A** local recurrence-free survival, **B** regional recurrence-free survival for patients stratified by the cutoff value of SUVmax value; **C** overall survival

The cumulative survival curves for the LH, HL, and HH groups were very close, but these curves were clearly separated from the survival curve for the LL group (*P* = 0.044; Fig. 3). The OS rate of the LL group significantly differed from those of the other 3 groups. Therefore, we reorganized all patients into 2 groups: a high-risk group consisting of the patients in the LH, HL, and HH groups, and a low-risk group consisting of the LL group patients. We found that the high-risk group had worse RRFS, LRFS, PFS, and OS rates than the low-risk group (all *P* < 0.05; Supplementary Figure S6).

### NTR and prognosis in high-risk group

We further analyzed the high-risk group patients. ROC curve analysis revealed that the optimal cutoff value of the NTR for predicting DMFS was 0.23 in the LH group, 2.35 in the HL group, and 1.29 in the HH group. The high-risk group patients were again reorganized into a low NTR group (NTR ≤ 0.23, ≤ 2.35, and ≤ 1.29 in the LH, HL, and HH groups, respectively) and a high NTR group (NTR > 0.23, > 2.35, and > 1.29 in the LH, HL, and HH groups, respectively).

**Fig. 4:**
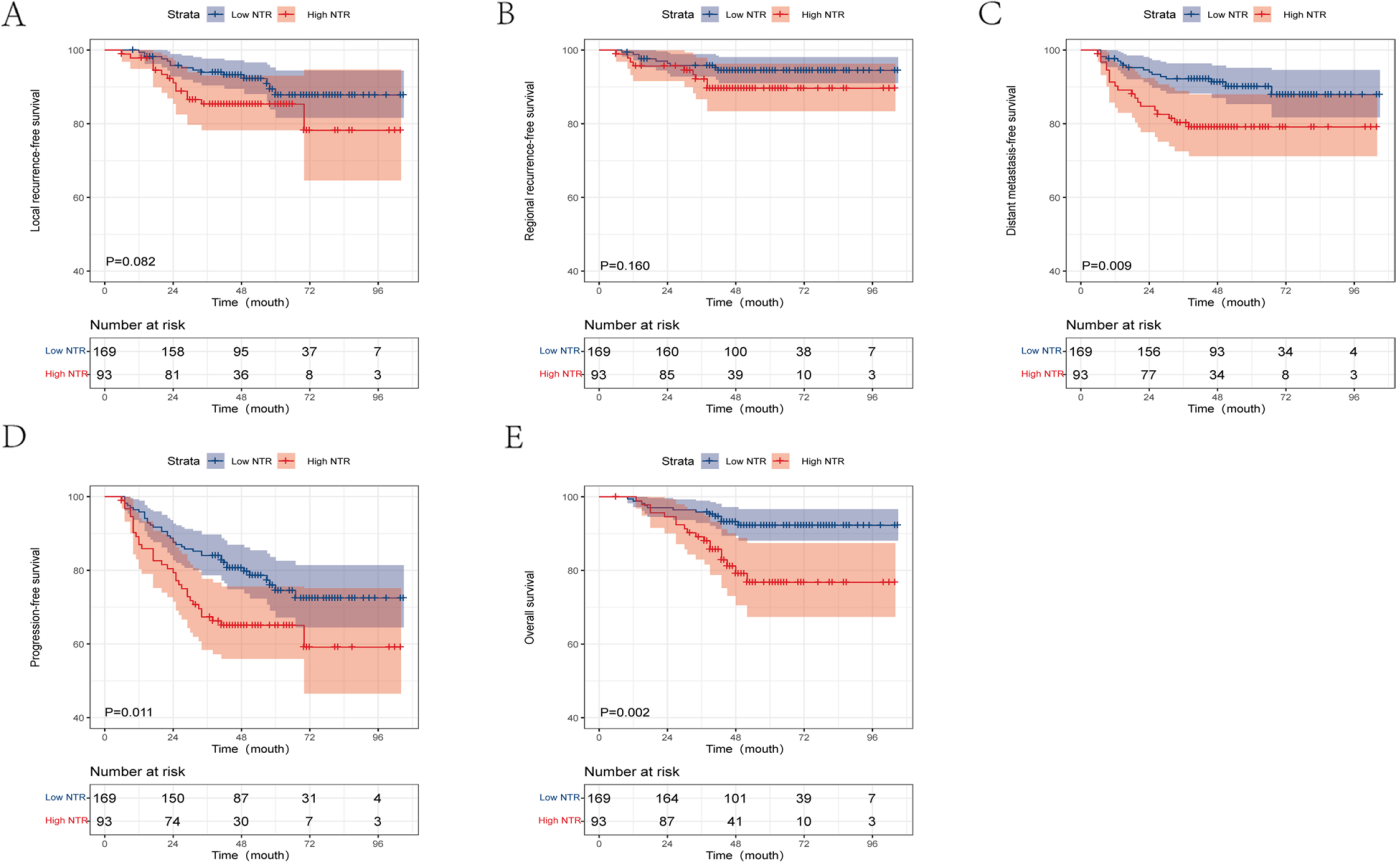
Kaplan-Meier by high NTR and low NTR group in high-risk patients. **A** local recurrence-free survival, **B** regional recurrence-free survival, **C** distant metastasis-free survival, **D** progression-free survival, and **E** overall survival

The 3-year LRFS, RRFS, DMFS, PFS, and OS rates in the low NTR vs. high NTR group were 94.1% *vs.* 86.0% (*P* = 0.082), 95.9% *vs.* 92.5% (*P* = 0.160), 92.3% *vs*. 80.6% (*P* = 0.009), 84.0% *vs.* 67.7% (*P* = 0.011), and 95.9 *vs.* 89.2% (*P* = 0.002), respectively. The OS, PFS, and DMFS rates were significantly worse in the high NTR group than in the low NTR group (all *P* < 0.005; Fig. 4).

Multivariate analysis with other known prognostic factors, including tumor stage (T stage), nodal stage (N stage), and age, revealed that the NTR was an independent prognostic factor for DMFS (HR = 2.037, 95% CI: 1.039–3.992, *P* = 0.038), PFS (HR = 1.636, 95% CI: 1.021–2.621, *P* = 0.041), and OS (HR = 2.543, 95% CI: 1.214–5.325, *P* = 0.013). The results of the multivariate analysis are summarized in Table 2.

**Table 2:** Multivariate analysis of LRFS, RRFS, DMFS, PFS and OS for NTR

Variables		Multivariate analysis	
		P	HR (95%CI)
Test for LRFS			
Age50	<50 vs. ≥50	0.594	1.223(0.583-2.564)
T	T1-T2 vs. T3-T4	0.021	2.889(1.170-7.135)
N	N0-N1 vs. N2-N3	0.726	0.877(0.421-1.828)
Group	Low NTR vs. High NTR	0.093	1.879(0.899-3.926)
Test for RRFS			
Age50	<50 vs. ≥50	0.680	1.218(0.477-3.112)
T	T1-T2 vs. T3-T4	0.417	0.682(0.270-1.720)
N	N0-N1 vs. N2-N3	0.008	5.352(1.542-18.58)
Group	Low NTR vs. High NTR	0.272	1.689(0.664-4.297)
Test for DMFS			
Age50	<50 vs. ≥50	0.258	1.472(0.753-2.879)
T	T1-T2 vs. T3-T4	0.036	2.261(1.055-4.848)
N	N0-N1 vs. N2-N3	<0.001	9.001(3.170-25.55)
Group	Low NTR vs. High NTR	0.038	2.037(1.039-3.992)
Test for PFS			
Age50	<50 vs. ≥50	0.020	1.748(1.091-2.799)
T	T1-T2 vs. T3-T4	0.029	1.760(1.059-2.926)
N	N0-N1 vs. N2-N3	<0.001	2.730(1.648-4.522)
Group	Low NTR vs. High NTR	0.041	1.636(1.021-2.621)
Test for OS			
Age50	<50 vs. ≥50	0.105	1.826(0.881-3.786)
T	T1-T2 vs. T3-T4	0.532	1.268(0.602-2.670)
N	N0-N1 vs. N2-N3	0.014	2.777(1.232-6.260)
Group	Low NTR vs. High NTR	0.013	2.543(1.214-5.325)

*Abbreviations*: *NTR* SUVmax-n/SUVmax-t ratio, Low NTR: NTR-LH < 0.23, NTR-HL < 2.35, and NTR-HH < 1.29; High NTR: NTR-LH ≥ 0.23, NTR-HL ≥ 2.35, and NTR-HH ≥ 1.29, *HR* hazard ratio, *CI* confidence interval

## Discussion

NPC has a better prognosis than other head and neck cancers. However, NPC patients with the same clinical stage and mode of clinical treatment may have different prognoses. The main causes of treatment failure in NPC are distant metastasis and local recurrence. Hence, the identification of predictors of metastasis or recurrence is of great interest because these could allow treatment to be tailored to the individual characteristics of the patient.

As a functional imaging technology, PET is a relatively new interdisciplinary technique for displaying the anatomy and morphology of lesions. ^18^F-FDG PET is based on the metabolic activity of the tumor and its parameters can reflect the biologic aggressiveness. SUVmax is the highest standardized uptake value within a volume of interest which reflects the part with the highest metabolic activity [15]. Recently, other functional and volumetric parameters, such as termed total lesion glycolysis (TLG), metabolic tumor volume (MTV) and SUVpeak also showed potential prognostic value [16, 17]. For instance, a prospective study proved that TLG was an independent prognosticator of OS in stage III–IVb NPC [18]. However, these volume-based parameters have not been sufficiently evaluated because the results were varied and controversial. Some researches declared that TLG and MTV did not show significant prognostic value for NPC [10, 11, 19]. Thus, SUVmax has been the most widely used parameter because the advantages of high accuracy, convenient measurement, and good repeatability.

As early as 2008, Lee et al. reported that SUVmax may predict disease-free survival, and that higher SUVmax may be useful for identifying patients requiring more aggressive treatment [20]. In 2015, Xiao et al. suggested that SUVmax at the primary site is a useful biomarker to predict distant metastasis in NPC patients treated with IMRT [21]. Subsequent studies by Jeong et al. and Cho et al. came to similar conclusions that the SUVs of the lymph nodes are important prognostic factors for distant metastasis [8, 11]. Consistent with the above studies, the present study showed that SUVmax reflects tumor aggressiveness and has prognostic importance in NPC. Additionally, our study investigated the potential link between the SUVs of the primary tumor and the lymph nodes by analyzing the NTR. The NTR has been reported to be strongly related to clinical outcomes and pathological characteristics in many tumor types such as esophageal carcinoma, endometrial carcinoma, and cervical carcinoma [22–24]. Chung et al. demonstrated that as the NTR increased, the risk of recurrence increased significantly in cervical carcinoma [24]. What’s more, there were strong correlations between NTR and lymphovascular space invasion, deep myometrial invasion, lymph node metastasis and high tumor grade (all *P* < 0.05) in gynecological oncology [23, 24]. That is to say, NTR may be a novel prognostic factor compensating the inherent limit of possible underestimation of SUV due to the partial volume effect. Hung et al. reported that the pretreatment NTR is a potential prognosticator for DMFS in NPC [9]. These findings suggest that the NTR is a novel marker for tumor aggressiveness, metastatic potential, and poor prognosis in NPC patients. We used different combinations of SUVmax-t and SUVmax-n to divide our total study population into 4 subgroups. The fact that the same cutoff of NTR be defined in different subgroups can be confusing even be incorrect in previous studies.

Considering the results of previous studies, we reviewed and analyzed the prognostic usefulness of SUVs in NPC. However, unlike previous studies, we took recurrence as an indicator of tumor aggressiveness. The 369 study patients were divided into 4 groups according to their SUVmax-t and SUVmax-n (LL, LH, HL, and HH groups). Comparative analysis of the groups showed that patients with a low SUVmax had better survival, suggesting that tumors with high ^18^F-FDG uptake could be more aggressive. Priority, more aggressive treatment or be followed up more closely, should be given to patients with a high SUVmax to improve their prognosis.

In the entire study cohort, we found no obvious correlation between SUVmax-t and SUVmax-n; however, both SUVmax-t and SUVmax-n increased with the T and N stage, respectively. This paper attempts to explore the reasons of tumor metastasis capacity from the aspects of tumor metastasis heterogeneity and the interaction between metastatic lymph node and primary lesion. The NTR is a good index that reflects the heterogeneity between metastatic lymph nodes and primary lesions. Moreover, this index has low inter-scanner variability. In our research study, a high NTR was associated with significantly worse DMFS and OS rates, suggesting that heterogeneity in the pretreatment ^18^F-FDG uptake between the lymph nodes and primary lesion is a strong indicator of tumor invasion and metastasis. This indicates that the NTR is a potential prognostic marker of tumor aggressiveness, metastatic potential, and poor prognosis in NPC patients with high SUVmax. By further analyzing the NTRs in different subgroups, we attempted to avoid cross-sensitization of cutoff points in different subgroups. The only cutoff points could create cross-risk and mix-ups in different subgroups. We hoped to ensure that clinicians could effectively differentiate prognostic risk between patients with different levels of heterogeneity in SUVmax between the metastatic lymph nodes and primary tumor.

High SUVmax-t was associated with local recurrence, while high SUVmax-n was associated with regional recurrence. Fei et al. found that in T4 NPC patients with a residual primary lesion after radical IMRT, a boost dose provides satisfactory tumor control with tolerable toxicities [25]. Yeh et al. recommended boosting irradiation to the neck for NPC patients with positive lymph nodes in order to achieve good regional control [26]. Further investigation is required to determine if local or regional control can be improved by increasing the irradiation dose to the target volume according to the SUV. Since our results showed that high NTRs are associated with significantly worse DMFS and OS rates, more aggressive systemic treatment is justified for such patients with high NTRs. Zong et al. demonstrated that in high-risk NPC patients, maintenance S1 chemotherapy following IMRT resulted in superior survival to that of patients treated without S1 chemotherapy [27]. A phase-2 multi-institutional trial (NCT00408694) reported that the addition of bevacizumab to standard chemoradiation treatment for patients with NPC is feasible, and might delay the progression of subclinical distant disease [28]. You et al. revealed that cetuximab or nimotuzumab in addition to concurrent chemoradiotherapy significantly improved 3-year OS (96.6% *vs.* 92.9%, *P* = 0.015) and 3-year DMFS (94.6% *vs.* 89.3%, *P* = 0.030) in patients with stage II–IVb NPC [29]. Xia et al. also supported that the addition of cetuximab to first-line chemoradiotherapy is associated with an improvement in DMFS in patients with locoregionally advanced NPC [30].

There were several limitations to the present study. First, as a retrospective study, treatment strategy and chemotherapy regimen among patients may be heterogeneous, which could be a selection bias and might influence the results. Second, SUV measurements may vary from different institutions depending on the differences in PET/CT protocols, scanners, and imaging analysis systems. This imposes limitations on reproducibility so the optimal cutoff value of ^18^F-FDG PET parameters in the present study may not consistently be the best in other researches. Well-designed prospective study is needed to confirm the present results and to determine the prognostic value of ^18^F-FDG PET in NPC.

## Conclusion

In summary, high SUVmax of the primary tumor or lymph node lesions is associated with local or regional recurrence of NPC. Patients with higher SUVmax had significantly worse survival, and should receive more aggressive treatment to improve their prognosis. Higher NTRs were associated with significantly worse DMFS and OS rates, suggesting that heterogeneity in the pretreatment ^18^F-FDG uptake between the primary tumor and lymph nodes is associated with high invasion and metastatic potential. Patients in different subgroups (LH, HL, and HH groups) required different cutoffs of NTR to avoid cross-sensitization. The above findings might help to identify patients who require boost irradiation to reduce the risk of recurrence or those who require more aggressive systemic treatment to reduce the risk of distant metastasis.

## Supplementary Information

**Additional file 1.** : Supplementary Table 1, Supplementary Table 2, Supplementary Figures S1-S6

## Data Availability

Data are available upon reasonable request. The data sets generated during and/or analyzed during the current study are available from the corresponding author on reasonable request.

## Abbreviations

SUVs: Standardized uptake values
NPC: Nasopharyngeal carcinoma
18F-FDG PET: 18F-fluorodeoxyglucose positron-emission tomography
SUVmax-t: SUVmax of the primary tumor
SUVmax-n: SUVmax of the regional lymph nodes
NTR: SUVmax-n/SUVmax-t ratio
OS: overall survival
DMFS: distant metastasis-free survival
PFS: progression-free survival
HR: hazard ratio
AJCC: The American Joint Committee on Cancer
IMRT: intensity-modulated radiotherapy
LRFS: local recurrence-free survival
RRFS: regional recurrence-free survival
ROC: receiver operating characteristic
CIs: confidence intervals
AUC: under the curve
