# Supplementary material for: Reflecting on the utility of standardized uptake values on 18F-FDG PET in nasopharyngeal carcinoma
Source: BMC Cancer. 2022 May 5;22:495. doi: 10.1186/s12885-022-09626-w (PMC9069730; doi:10.1186/s12885-022-09626-w)

# Appendix A. Supplementary material

**Supplementary Table 1.** Patient characteristics (n = 369).

| Characteristic | N | % |
| --- | --- | --- |
| Sex |  |  |
| Male | 269 | 72.9 |
| Female | 100 | 27.1 |
| Age(y) |  |  |
| Median (range) | 47(19-70) |  |
| ≥50 | 221 | 59.9 |
| <50 | 148 | 40.1 |
| Tumor category |  |  |
| T1 | 89 | 24.1 |
| T2 | 88 | 23.8 |
| T3 | 126 | 34.1 |
| T4 | 66 | 17.9 |
| Node category |  |  |
| N0 | 54 | 14.6 |
| N1 | 146 | 39.5 |
| N2 | 90 | 24.3 |
| N3 | 79 | 21.4 |
| Clinical stage |  |  |
| I | 27 | 7.3 |
| II | 67 | 18.2 |
| III | 136 | 36.9 |
| IVa | 139 | 37.6 |
| Posttreatment EBV DNA |  |  |
| Undetectable | 99 | 26.8 |
| Detectable | 270 | 73.2 |
| SUVmax-t |  |  |
| Mean ± SD | 9.2±5.2 |  |
| SUVmax-n |  |  |
| Mean ± SD | 6.8±5.8 |  |

**Supplementary Table 2.** Multivariate analysis of LRFS, RRFS, DMFS, PFS and OS for SUVmax.

| Variables |  | Multivariate analysis | | |
| --- | --- | --- | --- | --- |
|  |  |  | P | HR (95%CI) |
| Test for LRFS |  |  |  |  |
| Age50 | <50 vs. ≥50 |  | 0.596 | 1.202(0.610-2.368) |
| T | T1-T2 vs. T3-T4 |  | 0.018 | 2.687(1.187-6.079) |
| N | N0-N1 vs. N2-N3 |  | 0.123 | 1.770(0.856-3.661) |
| SUVmax-t | <7.5vs. ≥7.5 |  | 0.005 | 3.741(1.489-9.396) |
| SUVmax-n | <6.9vs. ≥6.9 |  | 0.003 | 0.269(0.114-0.637) |
| Test for RRFS |  |  |  |  |
| Age50 | <50 vs. ≥50 |  | 0.444 | 1.411(0.585-3.408) |
| T | T1-T2 vs. T3-T4 |  | 0.558 | 0.753(0.292-1.941) |
| N | N0-N1 vs. N2-N3 |  | 0.014 | 4.954(1.392-17.63) |
| SUVmax-t | <7.5vs. ≥7.5 |  | 0.541 | 0.739(0.280-1.951) |
| SUVmax-n | <6.9vs. ≥6.9 |  | 0.033 | 3.238(1.103-9.505) |
| Test for DMFS |  |  |  |  |
| Age50 | <50 vs. ≥50 |  | 0.270 | 1.384(0.777-2.465) |
| T | T1-T2 vs. T3-T4 |  | 0.038 | 1.954(1.039-3.678) |
| N | N0-N1 vs. N2-N3 |  | <0.001 | 9.634(3.993-23.24) |
| SUVmax-t | <7.5vs. ≥7.5 |  | 0.413 | 0.763(0.399-1.459) |
| SUVmax-n | <6.9vs. ≥6.9 |  | 0.710 | 0.888(0.475-1.660) |
| Test for PFS |  |  |  |  |
| Age50 | <50 vs. ≥50 |  | 0.020 | 1.643(1.083-2.494) |
| T | T1-T2 vs. T3-T4 |  | 0.022 | 1.698(1.080-2.672) |
| N | N0-N1 vs. N2-N3 |  | <0.001 | 3.720(2.288-6.050) |
| SUVmax-t | <7.5vs. ≥7.5 |  | 0.461 | 1.195(0.744-1.917) |
| SUVmax-n | <6.9vs. ≥6.9 |  | 0.306 | 0.785(0.493-1.248) |
| Test for OS |  |  |  |  |
| Age50 | <50 vs. ≥50 |  | 0.066 | 1.903(0.958-3.782) |
| T | T1-T2 vs. T3-T4 |  | 0.826 | 1.085(0.525-2.242) |
| N | N0-N1 vs. N2-N3 |  | 0.002 | 3.850(1.658-8.942) |
| SUVmax-t | <7.5vs. ≥7.5 |  | 0.228 | 1.637(0.734-3.648) |
| SUVmax-n | <6.9vs. ≥6.9 |  | 0.829 | 1.086(0.515-2.291) |

Abbreviations: SUVmax-t, standardized uptake value of the primary tumor; SUV max-n, the highest standardized uptake value of neck lymph nodes; DMFS, distant metastasis-free survival; LRFS, local recurrence‐free survival; RRFS, regional recurrence-free survival; DMFS, distant metastasis-free survival; PFS, progression-free survival; OS, overall survival.

**Figure Legends**

**Supplementary Figure S1.** Correlation between SUVmax-t and SUVmax-n.


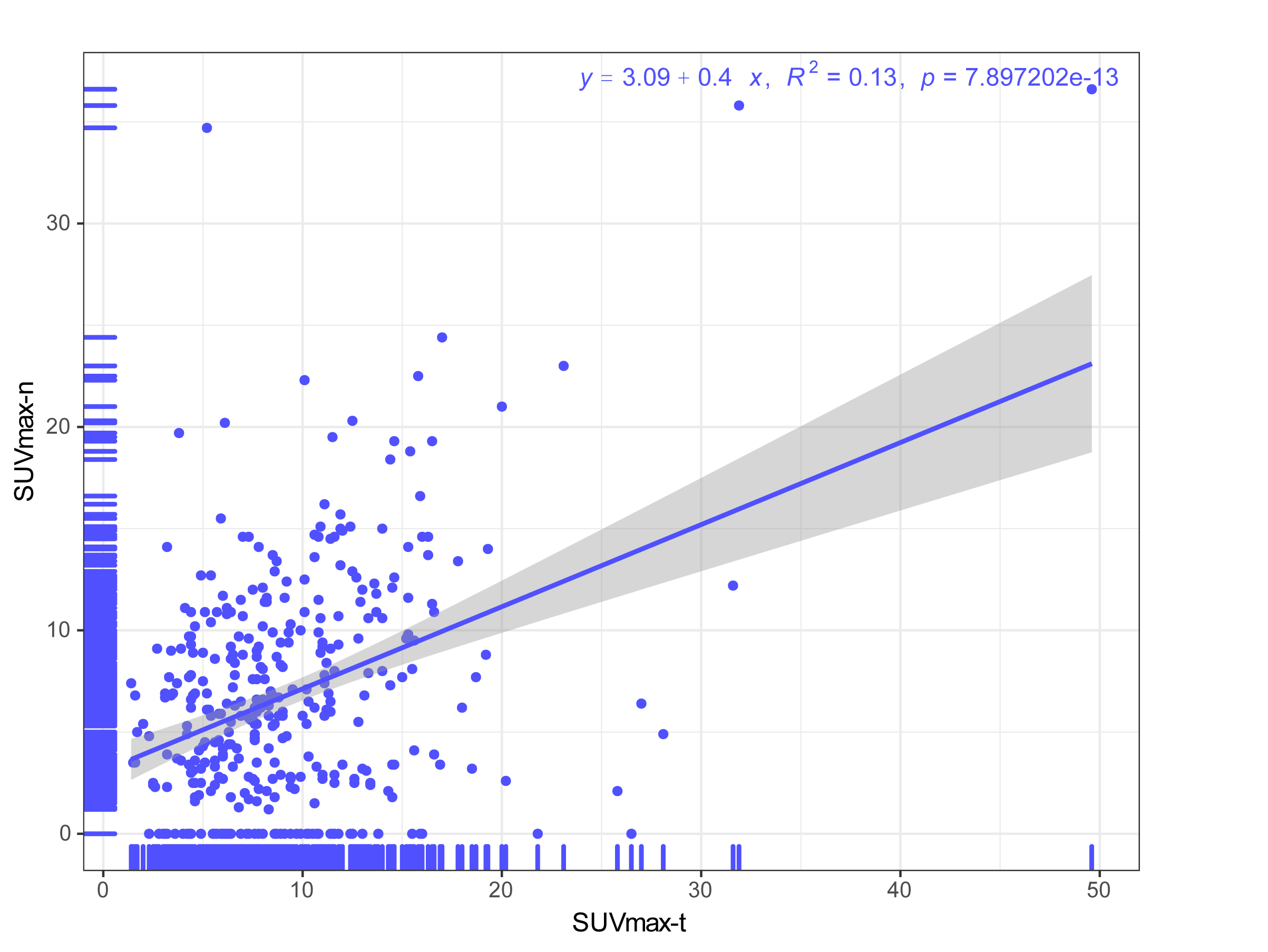


**Supplementary Figure S2.** Presentative cases for LL group, low SUVmax-n (≤6.9) and low SUVmax-t (≤7.5) : Patient A, SUVmax-t=3.7, SUVmax-n=3.7, within 87 months of follow-up, did not appear local and regional recurrence or distant metastasis.

**
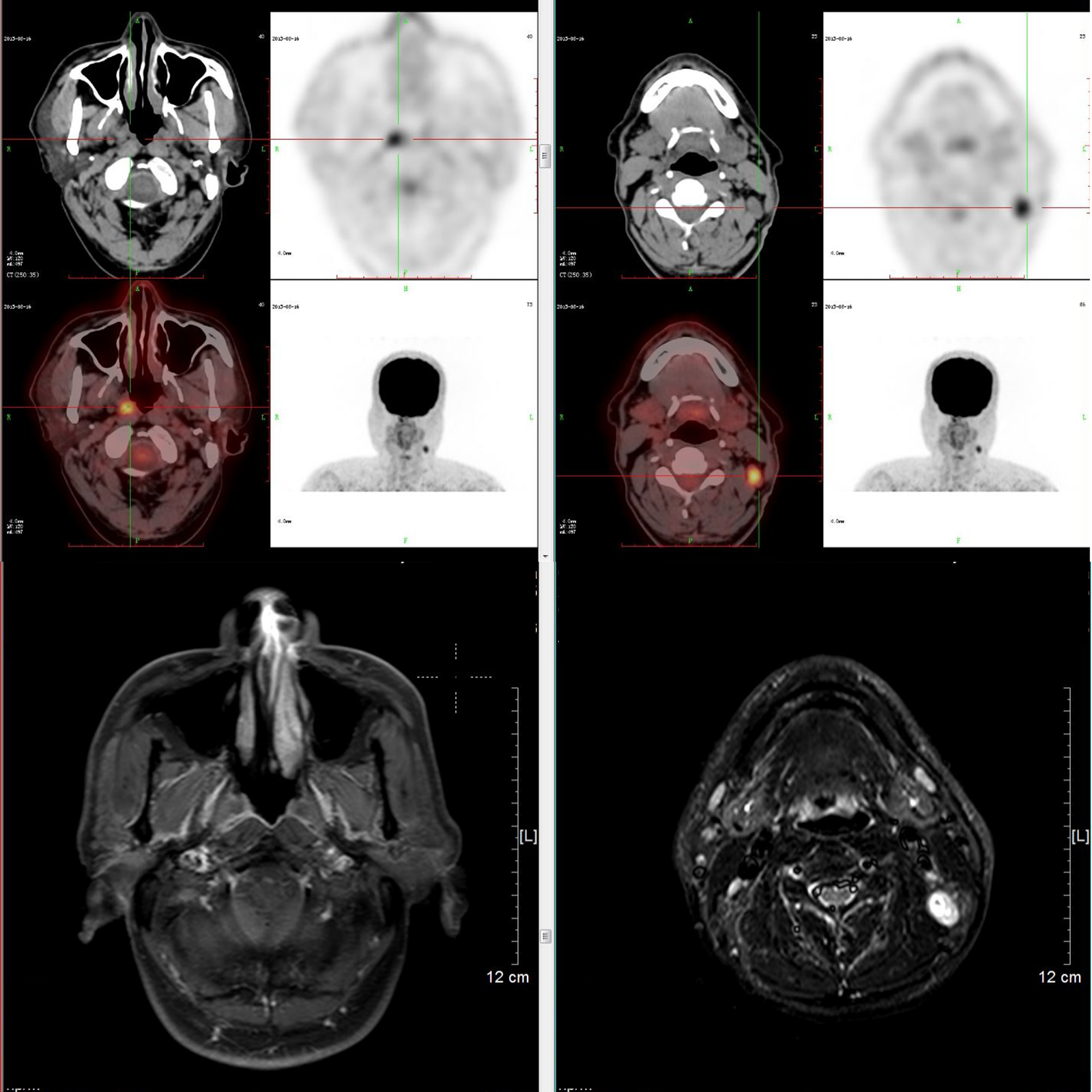
**

**Supplementary Figure S3.** Presentative cases for LH group, low SUVmax-n (≤6.9) and high SUVmax-t (>7.5) : Patient B, SUVmax-t=15.6, SUVmax-n=4.1, appear local recurrence in 17 months and distant metastasis in 18 months from diagnosis.

**
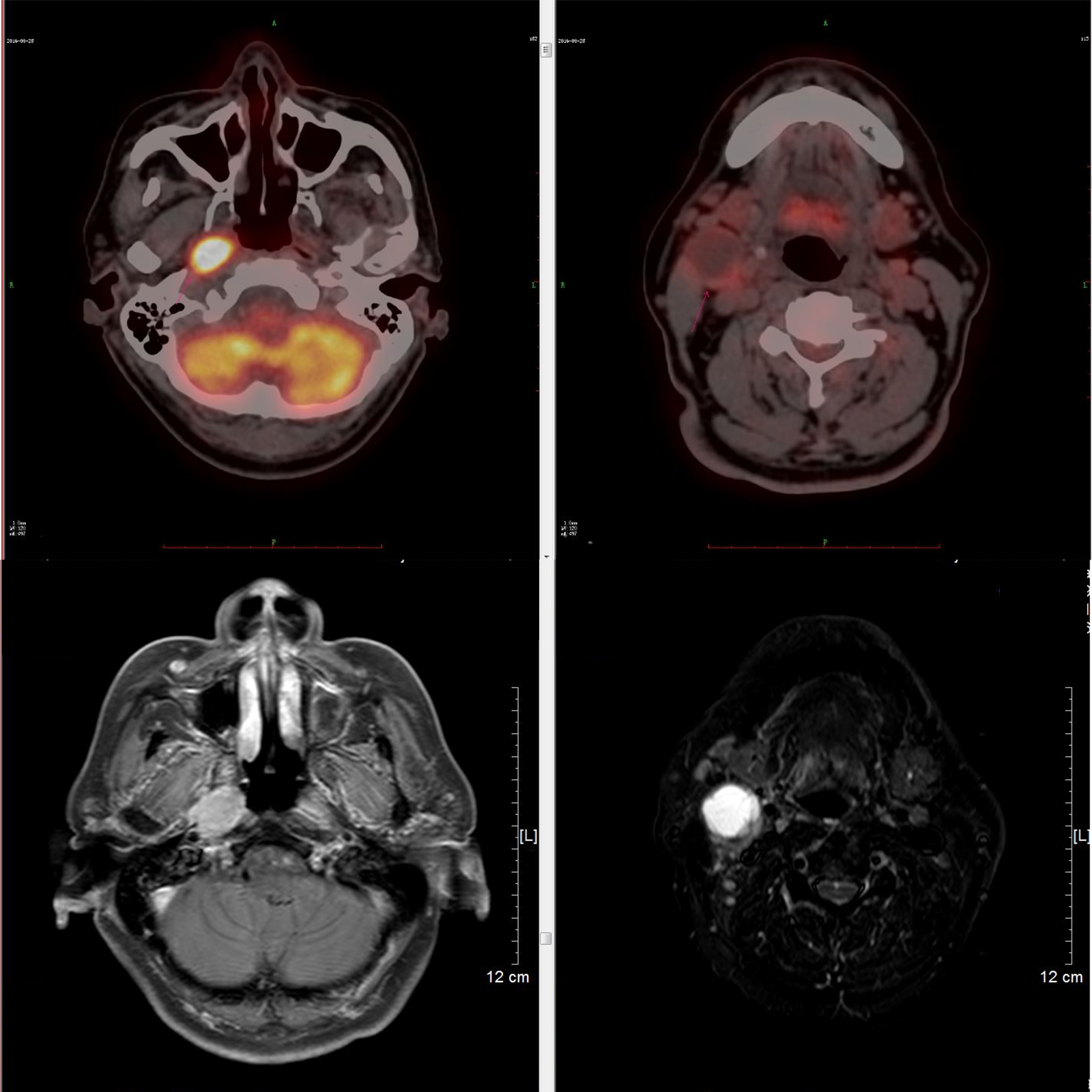
**

**Supplementary Figure S4.** Presentative cases for HL group, high SUVmax-n (>6.9) and low SUVmax-t (≤7.5) : Patient C, SUVmax-t=2.7, SUVmax-n=9.1, appear regional recurrence in 12 months and distant metastasis in 21 months from diagnosis.


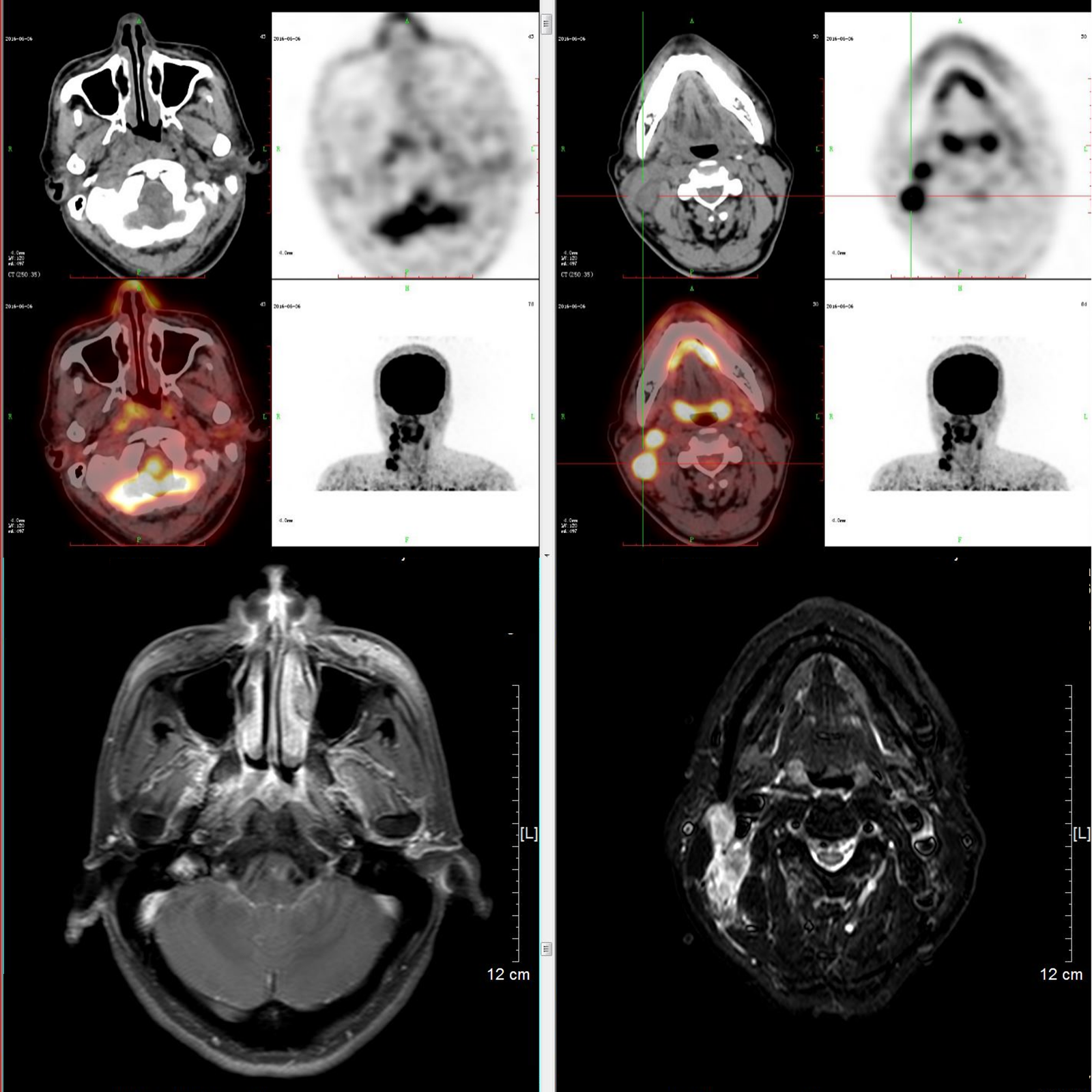


**Supplementary Figure S5.** Presentative cases for HH group, high SUVmax-n (>6.9) and high SUVmax-t (>7.5) : Patient D, SUVmax-t=14.6, SUVmax-n=12.6, appear local and regional in 14 months from diagnosis.


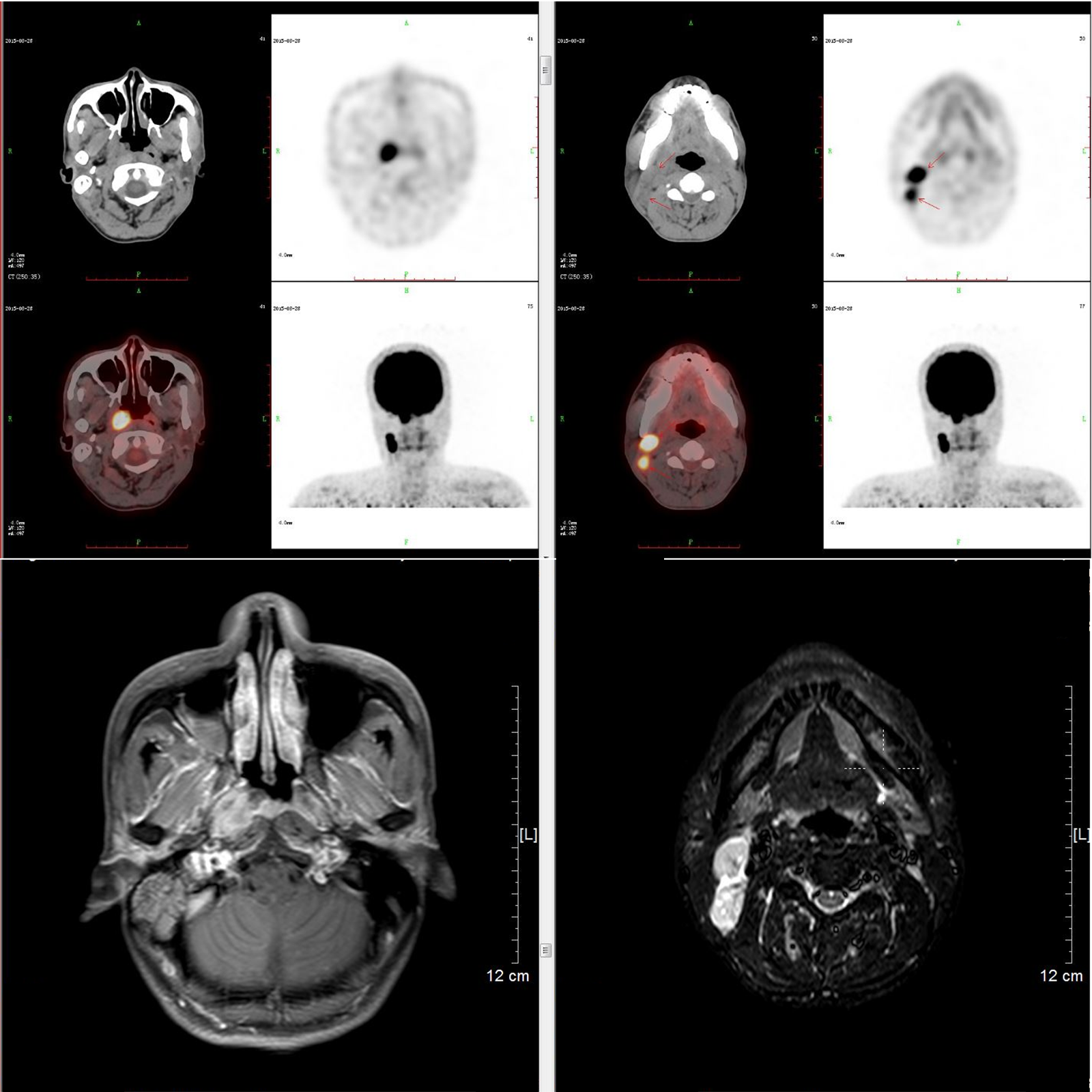


**Supplementary Figure S6.** Kaplan-Meier curves of (A) locoregional recurrence-free survival, (B) regional recurrence-free survival, (C) distant metastasis-free survival, (D) disease-free survival, and (E) overall survival for patients stratified by high NTR and low NTR group.


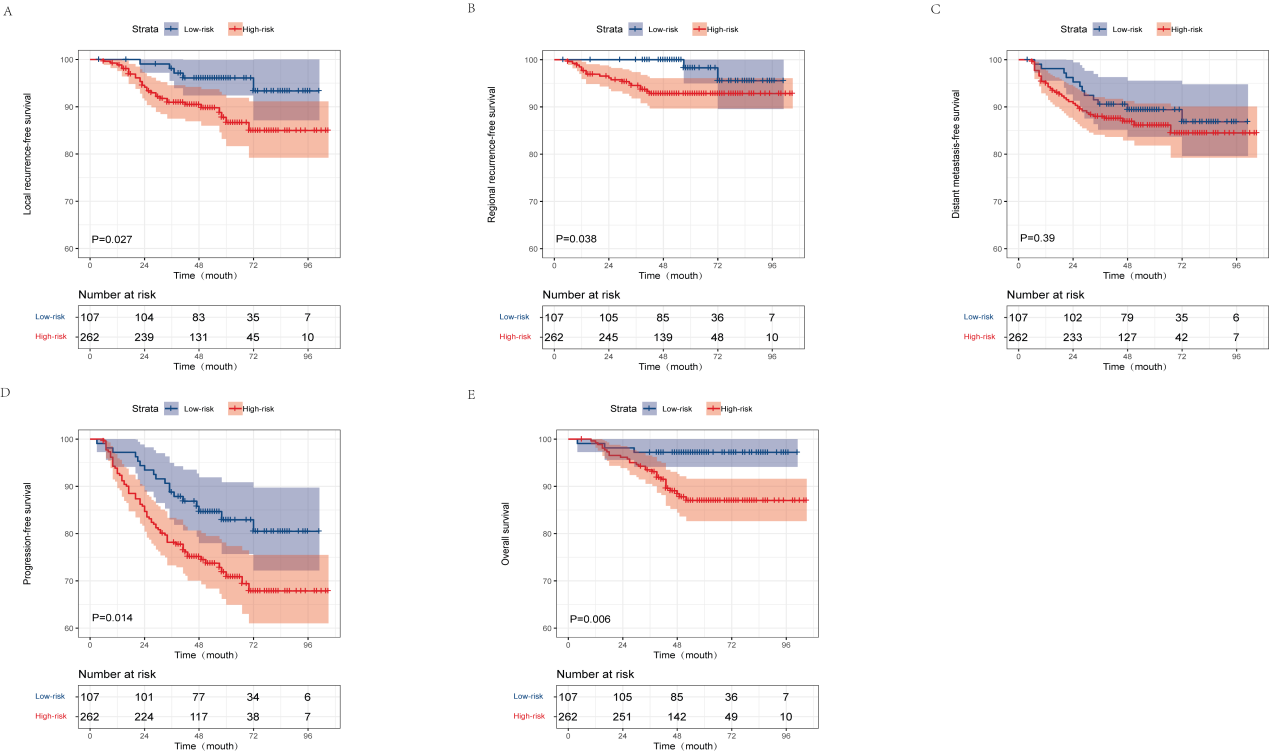

Supplement: Supplementary file 1 — Additional file 1. : Supplementary Table 1, Supplementary Table 2, Supplementary Figures S1-S6 [file 12885_2022_9626_MOESM1_ESM.docx]
